# Baltimore Academy of Medicine

**Published:** 1880-02

**Authors:** Eugene F. Cardell

**Affiliations:** Reporting Secretary


### Reports of Societies.

### BALTIMORE ACADEMY OF MEDICINE.

BALTIMORE, Dec. 16, 1879.

Dr. A. B. Arnold read a paper entitled "Obscure Affections of the Nervous System." Reference was particularly made to hysteria, spinal irritation and neuro-asthenia. The obscurity of these affections relates not only to their etiology and morbid anatomy, but also often to their diagnosis and symptomatology. Cases in illustration of various phases of hysteria, drawn from both sexes, were related. The connection of hysteia with any particular organ was regarded with disfavor, and the view advanced that it was rather an exaggerated degree of that condition which we call nervousness. Alcoholic stimulants were considered the most potent agent for the relief of hysterical affections, but the danger from their use was fully pointed out. Valerian and assafœtida have only a negative value.

Dr. P. C. Williams said that both alcohol and chloral should be avoided in hysteria. He had seen the habitual use of both originate from their employment for the relief of hysteria. Chloralism is as distressing as intemperance. The disease involves no danger to life, and we should hesitate to subject our patients to evils worse than the condition we seek to relieve.

Dr. H. P. C. Wilson agreed with the previous speakers as to the effects and dangers of alcohol. Few patients consult him for uterine disease of long standing who are not addicted to the use of alcohol, opium or chloral, the frequency of use corresponding with the order in which these agents are here named. He follows one rule in commencing the treatment of all cases of uterine disease: To stop short the use of any of these to which the patient may be addicted, and never to order either for a hysterical woman, unless its use be imperative, and then by the bowel or hypodermically, so that she will not know what she is using.

Dr. Wilson said that he never sees hysteria in either sex, without some organic cause, which is generally referable to the genital organs.

We cannot manage uterine disease in private practice as we can in hospitals, because it is impossible in the former case to break off the bad habits of patients, without which cure is not to be expected.

Dr. Arnold said it would greatly simplify the pathology of hysteria if we could refer it to one organ of the body, but this is manifestly impossible, as the variety and disconnectedness of the symptoms show. How can we establish a relation between these different phenomena and the genital system? They point rather to some general or systemic cause. The strain upon mind and nervous system (as from grief, etc.) is greater than that upon the genital organs. Now, items bearing upon this part of the patient's history are precisely those which we are not apt to get, whereas we readily find womb flexions, etc. If hysteria be referable to the generative system, how account for those cases of hystero-epilepsy after the menopause, reported by Charcot, or for those undoubted hysterical manifestations occurring before puberty? Moreover cases of uterine disease without hysteria are common enough. If it depend upon conditions referable to the womb, then the symptoms must be entirely reflex in character. But we must go further back in order to seek the pathology. All that we are justified in saying at present is that it has a number of exciting causes, but these alone are incapable of producing it except in the presence of a certain constitutional nervous peculiarity. In some women no exciting cause will produce it, but if this constitutional condition be once brought on, the womb troubles may act as exciting causes, and they may possibly be the most frequent exciting cause of all. Many neurologists entirely ignore the uterine theory. Nothing will take the place of opium, alcohol and chloral in the treatment.

Dr. Wilson replied that these agents are only palliative, not curative, and require repetition.

He did not wish to be understood as implying that infections of the ovary and womb are the only causes, since it occurs in the male sex.

Hysteria is a morbid state of mind, and only one form of nervous trouble reflected from the sexual organs, which are the centre of nervous sympathy in woman. It was the organic cause of hysteria to which he would call attention.

Dr. J. Carey Thomas said that it becomes often the duty of the physician to withhold from the patient a knowledge of the existence of disease, instancing the views of Flint with reference to heart murmurs. The only effect of such information in many persons of strong imagination and easily excited fears would be to produce a permanent morbid condition of the mind, rendering life miserable and aggravating perhaps rather than benefiting the possibly slight disease present. A case of syphilophobia was cited, in a young man who had been in the hands of a quack, to show the powerful and permanent impression upon the mind of information, even when false, given to patients.

Dr. Williams thought that young girls had better be left to suffer with many uterine diseases, than to have their minds rendered morbid and melancholy by the examinations and treatment necessary for their relief and cure.

Hysteria is at most only a reflex trouble, no matter where the exciting cause is. Hysterical symptoms are not the disease. We ought to go back and give name to the primitive organic trouble upon which these phenomena depend, and of the development of which they may be only the first appreciable signs. In the study of this subject specialists lay undue stress upon their special departments.

There are times at which we are bound to prescribe the objectionable remedies named, although ordinarily the speaker would oppose their use. Many women become hysterical in order to get the stimulant, and the only way to cure these is to let them understand they are not to have it.

Dr. Thomas requested the experience of the members of the Academy as to the length of the stage of incubation in diphtheria.

Dr. Williams related a case in an adult where it seemed to be nine days.

Dr. Arnold was under the impression that it was rather short. He once saw seven persons in one house take diphtheria, all within a period of ten days.

Dr. D. I. McKew said there were so many sources of contagion in a city that the point is one difficult to decide here; he thought that physicians practicing in the country had the best opportunities for settling it.

Dr. Jas. Steuart thought the susceptibility of the disease differs, and that during an epidemic the period of incubation is probably much shorter than where the disease is conveyed from one person to another. He related cases in which the duration seemed to be five and ten days respectively.

Dr. Cordell related a case of adherent placenta after miscarriage in the third month. There was profuse hemorrhage, estimated at about one gallon. The placenta had to be scraped off piecemeal from its seat of attachment by the fingers, the entire hand being introduced into the va gina, under chloroform, for this purpose. There was no further hemorrhage, and the patient slowly recovered from the extreme exhaustion.

---

BALTIMORE, Jan. 7th, 1880.

*Hysterical Mydriasis Simulating and Treated for Amaurosis.*—Dr. Chisolm related the case of a young lady, aged 21, who applied to him for treatment on account of loss of sight of one eye, which, according to her statement, originated in a fall from her horse nine years before, but which grew much worse during a recent and violent attack of gastric fever accompanied by vomiting of blood, from which she had suffered four months previous to her visit. She had lost entirely the power to distinguish objects, and could only discern light. She was unable to count the fingers held before her. She presented a good condition, notwithstanding her declaration that she ate but little, and slept very badly. She had been under the care of several physicians, for many months past, for what they had pronounced as amaurosis. On careful examination, the only abnormality to be discovered was dilatation of the pupil of the affected eye.

Suspecting that the defective vision was feigned and unreal, the patient was required to look through a stereoscope at two pictures—a bird and a cage—one placed before each eye. Now, if the sight of one eye were lost, the picture placed in front of it would of course not be seen. On being asked what she saw, she replied, "a bird in a cage," showing that she had good sight in both eyes.

*Modification of the Operation of Section of the Optic and Ciliary Nerves for Sympathetic Opthalmia.*—Dr. Chisolm also mentioned a simplification of this operation, which consists in an incision through the conjunctiva, from the inner edge of the cornea to the caruncula. Access is thus gained to the bottom of the orbit, and the necessity of dividing the internal rectus and stitching it again to its point of attachment to the ball (an essential feature of the old method) avoided.

*Aneurism of Ascending Portion of Arch of Aorta.* Dr. Tiffany reported a case in which a small sacculated aneurism involved the commencement of the aorta, one inch above the aortic valves. It was situated partly within the pericardium, and only became visible on opening this. The patient, a robust teamster, aged 45, had only been under observation one week, and death occurred from an intercurrent affection. Three weeks before coming under treatment this patient began to suffer from occasional dyspnœa, with swelling and cyanosis of the face (especially on first rising in the morning.) He complained of slight pain in the right mammary region, the veins in the same situation and also over the liver were dilated, there was slight dulness at the base of the right lung in front, and the right half of the chest expanded three-fourths inch more than the left. The respiratory murmur was everywhere normal, there was no bruit discoverable on auscultation, the pulse was 76 and alike on the two sides, the voice was natural and there was no cough. The man could assign no cause for the trouble. During the attacks of dyspnœa the only way he could obtain any relief was by lying in a prone position over a bed with his head hanging down over the edge. The diagnosis was probable aneurism of the commencement of the aortic arch, projecting backwards, and to the right. The treatment ordered was veratrum viride, recumbency, restricted and dry diet, and avoidance of fluids, but of course a sufficient time had not elapsed to test the virtue of these measures. The dyspnœa and cyanosis were found to be due to pressure of the tumor on the ascending cava vein, and recurrent laryngeal nerve.

Dr. Cordell referred to the diagnostic importance of persistent pain in the spine in cases of aneurism of the aorta. His attention was first directed to the great value of this symptom by a paper of Dr. Lidell, which appeared about twelve years ago in the *Am. Jour. of Med. Sciences.* In reliance chiefly upon it, he had been enabled to make a diagnosis in at least two cases, which otherwise would have been exceedingly obscure. Its characteristic features are its obtuseness, its persistence and its fixedness. The significance of these features is of course to be sought in other considerations (positive and negative) relating to the case, upon which their correct interpretation depends.

The following case was related in illustration: He was summoned, in great haste, to see a man of about fifty, who was suddenly seized with alarming symptoms. On arrival he found the man lying on the bed dead. The family and friends were seated around, and not suspecting the fatal issue. On inquiry it was learned that he had been complaining for some time of slight chest trouble, and of a pain in the spine between the scapalæ, constant but never severe, and that these symptoms had not prevented his attention to his business—that of a retail merchant. A pretty confident diagnosis of aneurism of the arch of the aorta was expressed, which the post mortem shortly after verified.

Dr. Tiffany said that pain in the spine could only occur as a result of erosion of the vertebræ. He illustrated by a diagram the relative position of the aneurism and spinal nerves, to show that the former would not only have to pass around the bodies of the vertebræ to reach the seat of the latter, but that the latter emerged from the posterior surface of the vertebræ, and then passed outwards behind the ribs, which effectually protected them from pressure. Dr. Tiffany said that he averaged about two post mortem examinations of aortic aneurisms a year.

EUGENE F. CARDELL, M.D.,
Reporting Secretary.

